# Advances in the Study of Age-Related Macular Degeneration Based on Cell or Cell-Biomaterial Scaffolds

**DOI:** 10.3390/bioengineering12030278

**Published:** 2025-03-11

**Authors:** Ziming Li, Zhiyong Hu, Zhixian Gao

**Affiliations:** School of Public Health, Binzhou Medical University, Yantai 264003, China; ming13512006301@163.com (Z.L.); huzhiyong23@163.com (Z.H.)

**Keywords:** age-related macular degeneration, retinal pigment epithelium, stem cells, tissue engineering

## Abstract

Age-related macular degeneration (AMD), a progressive neurodegenerative disorder affecting the central retina, is pathologically defined by the irreversible degeneration of photoreceptors and retinal pigment epithelium (RPE), coupled with extracellular drusen deposition and choroidal neovascularization (CNV), and AMD constitutes the predominant etiological factor for irreversible vision impairment in adults aged ≥60 years. Cell-based or cell-biomaterial scaffold-based approaches have been popular in recent years as a major research direction for AMD; monotherapy with cell-based approaches typically involves subretinal injection of progenitor-derived or stem cell-derived RPE cells to restore retinal homeostasis. Meanwhile, cell-biomaterial scaffolds delivered to the lesion site by vector transplantation have been widely developed, and the implanted cell-biomaterial scaffolds can promote the reintegration of cells at the lesion site and solve the problems of translocation and discrete cellular structure produced by cell injection. While these therapeutic strategies demonstrate preliminary efficacy, rigorous preclinical validation and clinical trials remain imperative to validate their long-term safety, functional durability, and therapeutic consistency. This review synthesizes current advancements and translational challenges in cell-based and cell-biomaterial scaffold approaches for AMD, aiming to inform future development of targeted interventions for AMD pathogenesis and management.

## 1. Introduction

Retinal degenerative diseases (RDDs), encompassing hereditary, acquired, and age-related etiologies, represent the principal cause of irreversible global blindness, accounting for over 70% of severe vision impairment cases worldwide [1]. Age-related macular degeneration (AMD) exemplifies a prototypical neurodegenerative pathology, with its dry (non-neovascular) and wet (neovascular) subtypes collectively projected to affect 288 million individuals by 2040 [2]. This indicated that AMD may cause major public health problems, such as impaired population health, increased economic stress on patients, and increased socioeconomic burden.

Damage or loss of retinal pigment epithelium (RPE) cells is a major cause of AMD. RPE cells are crucial for maintaining the health of photoreceptor cells, and their dysfunction or loss can lead to photoreceptor degeneration and vision loss. In dry AMD, RPE cell loss leads to geographic atrophy (GA), while in wet AMD, the disruption of RPE cell tight junctions can induce the overexpression of vascular endothelial growth factor (VEGF), triggering choroidal neovascularization (CNV) [3]. Current therapeutic strategies to mitigate disease progression in AMD include intravitreal delivery of pharmacologic agents (e.g., long-acting/extended-release formulations, combination therapies, and gene therapy vectors) and neuroprotective interventions targeting photoreceptor-RPE survival pathways [4,5,6], but they fail to address the core pathophysiology: irreversible depletion of RPE-photoreceptor units.

Emerging evidence positions cell replacement therapy as a transformative strategy to reconstitute the RPE. Seminal work by Fisher et al. established that RPE dysfunction precedes photoreceptor degeneration in AMD pathogenesis, with metabolic coupling collapse and oxidative stress inducing a feedforward cycle of neuroretinal atrophy [7,8]. This mechanistic understanding has fueled clinical advances in stem cell-derived RPE transplantation [9]. Zhang et al. performed the first GMP-compliant preclinical evaluation of human-induced pluripotent stem cell-derived retinal pigment epithelial cells (hiPSC-RPE) in a rodent model in China. The transplanted hiPSC-RPE demonstrated sustained survival (≥19 weeks) without teratoma formation or aberrant proliferation (*p* < 0.01 vs. controls) while maintaining functional photoreceptor rescue for 15 weeks, as evidenced by 38% improvement in electroretinogram (ERG) b-wave amplitudes [10].

Nevertheless, critical barriers persist; there are also limitations to the therapeutic efficacy and therapeutic efficiency of cell therapy by cell proliferation, differentiation, in vivo survival, migration, and integration [11]. The utilization of cell-biomaterial scaffolds for AMD therapy demonstrates significant advantages over direct subretinal injection of stem cell-derived RPE suspensions. Firstly, cell-biomaterial scaffolds can enhance cell retention compared to cell therapy. Chen et al. demonstrated that encapsulating neural stem cells (NSCs) within hydrogel scaffolds significantly enhanced NSC retention (82% vs. 24% in suspension controls) and improved functional recovery in traumatic brain injury models, as measured by 41% increased synaptic density in hippocampal regions [12]. Separately, cell-biomaterial scaffolds also can improve spatial organization. Muthusamy et al. engineered 3D-bioprinted microtissue constructs that induced targeted vascularization in specified anatomical regions, achieving 2.3-fold higher capillary density compared to random endothelial cell seeding approaches [13].

This article systematically reviews and summarizes the cutting-edge research progress in the field of AMD, primarily focusing on cell-based and cell-biomaterial scaffold-based RPE replacement approaches. Initially, we compare three major therapeutic approaches—antibody-based, gene-based, and cell-based strategies—and provide a detailed analysis of their characteristics. We emphasize the application of progenitor/stem cells for in vitro RPE differentiation and their functional validation in in vivo models. Furthermore, we examine the functional properties and advantages of cell-biomaterial scaffolds in facilitating stem cell differentiation into RPE and enhancing RPE proliferation. Finally, we address critical challenges in RPE replacement therapies and highlight recent breakthroughs in preclinical and clinical research.

## 2. Age-Related Macular Degeneration

The retina, a multilaminar photosensitive tissue situated posteriorly within the ocular globe, exhibits an average thickness of approximately 0.5 mm [14]. The RPE, a monolayer of terminally differentiated, polarized epithelial cells situated adjacent to the neural retina, orchestrates the visual cycle through retinoid isomerization while maintaining photoreceptor homeostasis and preserving outer retinal architectural integrity [15]. AMD arises from the irreversible degeneration of RPE and photoreceptor cells, culminating in permanent vision loss and representing the predominant etiology of blindness in the geriatric population [16]. AMD is clinically categorized into two subtypes: dry (atrophic/non-neovascular) and wet (neovascular/exudative) AMD. The dry form, accounting for the majority of cases, is pathologically defined by the accumulation of drusen deposits and progressive geographic atrophy (GA). Often involving the foveal region [17], this atrophic progression, driven by RPE degeneration and subsequent photoreceptor-RPE complex disintegration, culminates in advanced-stage disease marked by irreversible central vision loss upon encroachment of the central fovea [18]. The pathological transition from dry AMD to the neovascular (wet) subtype is mechanistically driven by hypoxia-induced dysregulation of angiogenic homeostasis, characterized by aberrant CNV secondary to Bruch’s membrane breakdown, compensatory VEGF overexpression, and inflammatory-mediated vascular endothelial activation [17,19,20]. CNV is a critical risk factor for subretinal fibrosis, a pathological hallmark of advanced neovascular age-related macular degeneration (nAMD), characterized by aberrant fibrotic scar tissue formation driven by chronic inflammation, recurrent vascular leakage, and dysregulated tissue repair [21,22].

## 3. Treatment of AMD

**Figure 1:**
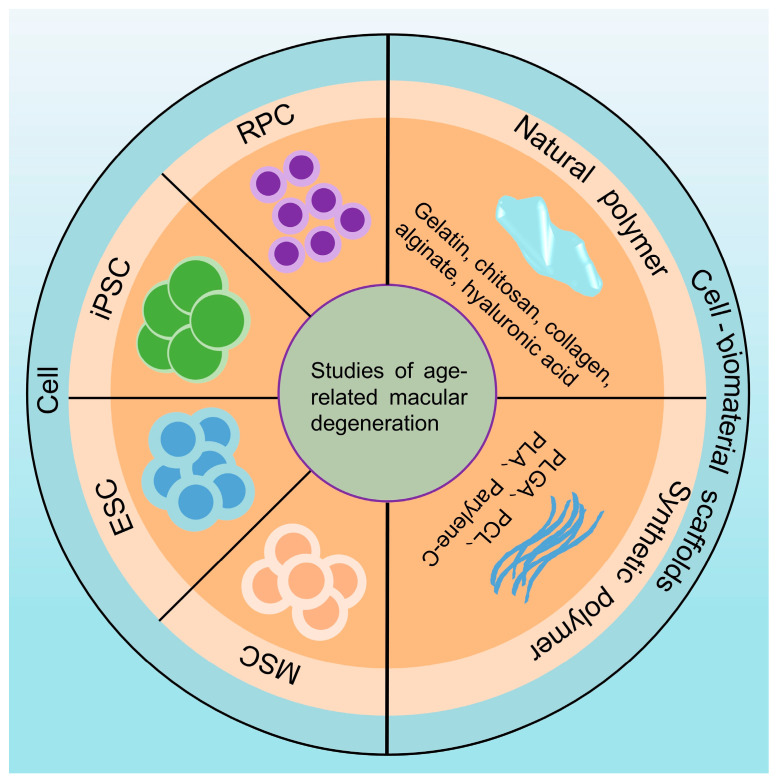
Schematic representation of the progress of cell-based and cell-biomaterial scaffolds for AMD.

AMD occurs as a result of a multifactorial (genetic, environmental, metabolic, and functional) combination of factors, and the pathogenesis is complex, but the underlying pathology is not well understood [23]. Current therapeutic modalities encompass pharmacologic agents (e.g., anti-VEGF biologics), gene therapies, and cell-based regenerative strategies. Table 1 summarizes recent advances in the three aforementioned therapeutic approaches. Among these, intravitreal anti-VEGF biologics have revolutionized the management of neovascular AMD, constituting the first-line standard of care in clinical practice [16]. Antibody studies targeting complement pathway inhibition are still ongoing, and there have been clinical trials demonstrating that avacincaptad pegol, a targeted complement C5 inhibitor, significantly reduced the progression of GA secondary to AMD, with rigorous assessment of both efficacy and safety endpoints [24]. Pegcetacoplan, a targeted complement C3 inhibitor, has emerged as the first FDA-approved therapy (2023) for GA secondary to AMD. Phase 3 clinical trial data demonstrated its capacity to significantly reduce the rate of lesion growth by 22–29% compared to sham treatment (*p* < 0.01), with a safety profile characterized primarily by mild-to-moderate ocular adverse events, including conjunctival hemorrhage in 13–20% of participants [25]. A recent phase I clinical trial published in The Lancet investigated the use of RGX-314 (now referred to as ABBV-RGX-314) for the treatment of nAMD. ABBV-RGX-314 is an adeno-associated virus serotype 8 vector that expresses an anti-VEGF-A antigen-binding fragment and has the potential for sustained VEGF-A inhibition following a single subretinal injection. The results indicated that subretinal delivery of ABBV-RGX-314 was generally well-tolerated, with no clinically significant immune responses observed. This therapy offers a novel approach for sustained VEGF-A inhibition in nAMD patients, with the potential to control exudation, maintain vision, and reduce the treatment burden after a single administration [26]. Heier and colleagues conducted a phase I clinical trial of JNJ-81201887 (JNJ-1887) gene therapy for AMD, administering a single intravitreal injection to treat GA secondary to dry AMD. The results demonstrated that JNJ-1887 exhibited favorable safety and tolerability in patients with GA secondary to dry AMD. Notably, in the high-dose cohort, the growth rate of GA lesions significantly decreased over 24 months, suggesting that this gene therapy may have the potential to slow the progression of GA. Further investigation is warranted to confirm the long-term efficacy and safety of JNJ-1887 in treating GA [27]. However, both antibody-based and gene therapies are intrinsically limited by their inability to restore the regenerative capacity of RPE. In contrast, cell-based and cell-biomaterials scaffold-based approaches focus on replacing degenerated RPE cells to stabilize long-term therapeutic outcomes in AMD. Consequently, the following content of this review will prioritize cell- or cell-biomaterial scaffold-mediated RPE replacement strategies. Figure 1 shows a schematic representation of the progress of AMD in the study of cell or cell-biomaterial scaffolds.

### 3.1. Cell-Level Studies of RPE Replacement

Cell-based therapeutic modalities represent a leading investigational strategy for degenerative retinopathies, including AMD, designed to achieve targeted RPE/photoreceptor replacement and paracrine-mediated neuroprotection to counteract disease progression. Neuroprotective interventions demonstrate efficacy in preserving RPE cells and photoreceptors during pre-apoptotic phases of AMD, yet their therapeutic utility diminishes in advanced-stage disease due to irreversible cellular loss and dysregulation of the retinal-choroidal microenvironment. AMD is pathologically characterized by progressive degeneration of the RPE and photoreceptor cells, culminating in irreversible central vision loss [50]. In response, regenerative strategies targeting RPE replacement have emerged as a focal point of preclinical and clinical investigation to restore retinal homeostasis. Rajendran et al. demonstrated that subretinal delivery of autologous RPE cell suspensions for AMD therapy was associated with localized inflammatory responses [51]. Furthermore, autologous grafts inherently retain the donor’s genetic predisposition to AMD pathogenesis, potentially perpetuating disease-associated pathways [52]. Therefore, current autologous RPE cell suspension therapies for AMD exhibit significant technical constraints, including limited proliferative capacity and inherent genetic vulnerability. In contrast, subretinally delivered progenitor/stem cell-derived RPE grafts demonstrate therapeutic superiority through: (1) allogeneic cell sources circumvent transmission of patient-inherited genetic susceptibilities; and (2) the pluripotency of stem cells enables the generation of various cell types, including RPE cells, holding greater therapeutic potential [11,52,53,54]. So many researchers have attempted to transform progenitor/stem cells with differentiation properties into healthy RPE, which can be transplanted to regenerate the damaged RPE [9,55,56].

Stem cell-based approaches have emerged as a focal point of investigation within regenerative medicine, demonstrating considerable therapeutic potential to restore retinal homeostasis, thereby positioning them as a viable strategy for vision restoration in patients with AMD [57,58]. Stem cells are defined as undifferentiated cells exhibiting three key defining properties: sustained self-renewal capacity, clonal expansion into pluripotent stem cell populations, and multipotent differentiation potential into specialized lineages [59,60]. Table 2 delineates the principal cellular sources under investigation for AMD therapy, including retinal progenitor cells (RPCs), embryonic stem cells (ESCs), induced pluripotent stem cells (iPSCs), and mesenchymal stem cells (MSCs), alongside their comparative advantages and limitations. As proposed by Coco-Martin et al. [61], these cells represent viable candidates for retinal cell transplantation, with directed differentiation protocols enabling their conversion into functional RPE, photoreceptors, or ganglion cells for clinical translation. A concise evaluation of these cellular platforms follows.

#### 3.1.1. RPCs

**Figure 2:**
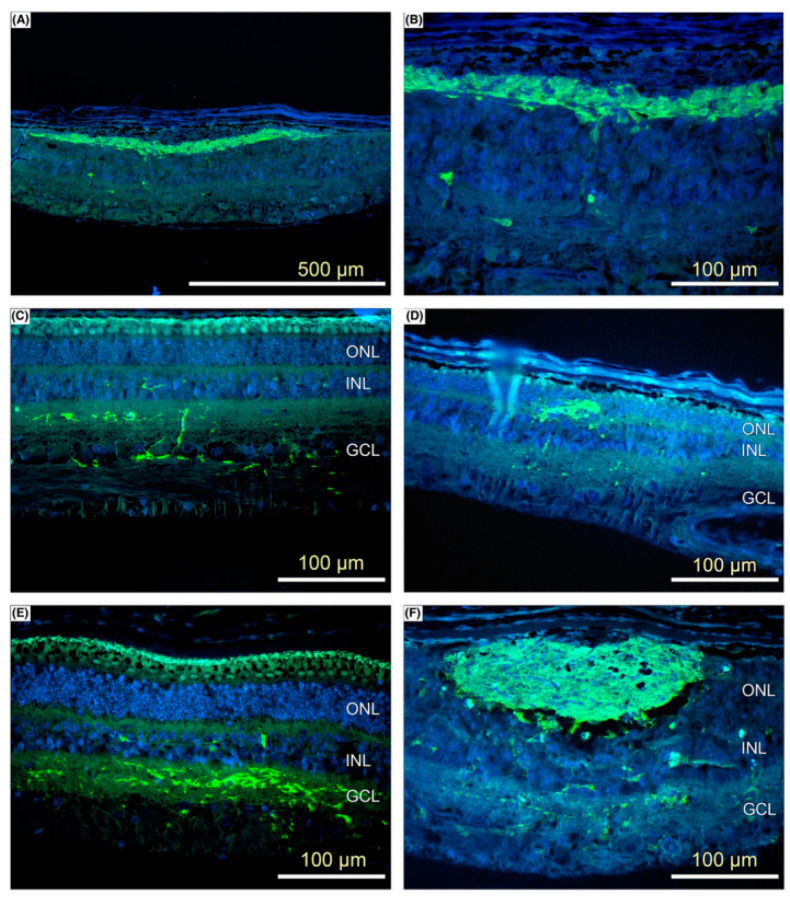
Photomicrographs of 5 μm retinal sections, captured at 3 weeks post-treatment, demonstrate the survival of implanted cells (green) at the 3-week time point (**A**–**E**). The green fluorescence indicates the presence of photoreceptor precursor cells (pRPCs), while the blue fluorescence represents Hoechst counterstaining for nuclear visualization. Additionally, images taken 24 h post-implantation (**F**) are included for comparative analysis. Reprinted from Ref. [68].

RPCs exhibit intrinsic advantages, such as a reduced risk of tumorigenesis and avoidance of immune rejection, rendering RPE or photoreceptors derived from RPC differentiation as viable and promising candidates for transplantation [68,69,70]. Figure 2 demonstrates that Abud et al. injected retinal progenitor cells (RPCs) into the porcine subretinal space, where photoreceptor precursor cells were observed three weeks post-injection, providing initial evidence of robust post-implantation cell survival [68]. However, their clinical translation is constrained by poor survival rates of transplanted RPCs and limited in vitro expansion capacity, necessitating the development of innovative cell culture techniques and advanced delivery methods to address these challenges [71].

#### 3.1.2. ESCs

**Figure 3:**
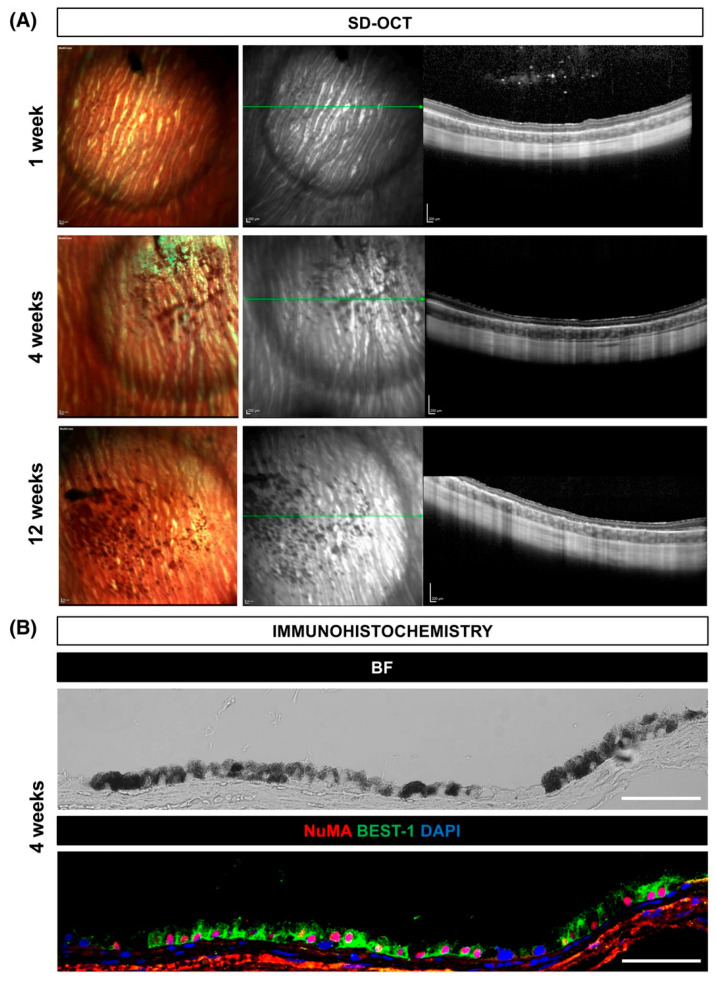
Subretinal engraftment of hESC-derived RPE cells in the albino rabbit model was evaluated through longitudinal multimodal imaging and histopathological analysis. (**A**) In vivo assessment utilizing multicolor confocal scanning laser ophthalmoscopy (cSLO) combined with spectral-domain optical coherence tomography (SD-OCT) demonstrated the structural integration of transplanted hESC-RPE cells at 1-, 4-, and 12-week postoperative intervals. The SD-OCT scan planes are demarcated by green reference lines. (**B**) Histological validation at the 4-week time point revealed successful RPE engraftment, as evidenced by bright-field microscopy and immunofluorescence staining for nuclear mitotic apparatus protein (NuMA) and bestrophin-1 (BEST-1), specific markers of RPE cellular integrity and functionality. Scale bars represent 200 μm for panel A and 50 μm for panel B. Reprinted from Ref. [77].

As pluripotent stem cells, ESCs possess the defining characteristics of self-renewal and multipotent differentiation capacity into diverse somatic lineages [72]. Hall et al. have found that ESC can differentiate into specific cell types [73]. Klimanskaya et al. pioneered the application of ESCs for RPE generation, establishing their utility as a foundational platform for RPE production [74]. Subsequent studies by Limnios et al. and Garcia et al. further validated the efficacy of human ESCs (hESCs) in generating functional human RPE monolayers with structural and phenotypic fidelity [75,76]. Figure 3 illustrates that Petrus et al. performed subretinal transplantation of human embryonic stem cell-derived RPE cells into albino rabbits and demonstrated that the in vivo integration of RPE cells led to the expression of specific markers associated with structural integrity and functionality [77]. ESCs demonstrate significant potential for retinal lineage differentiation; however, their clinical translation is constrained by ethical controversies, alloimmune rejection risks, and inherent tumorigenic potential [78].

#### 3.1.3. iPSCs

**Figure 4:**
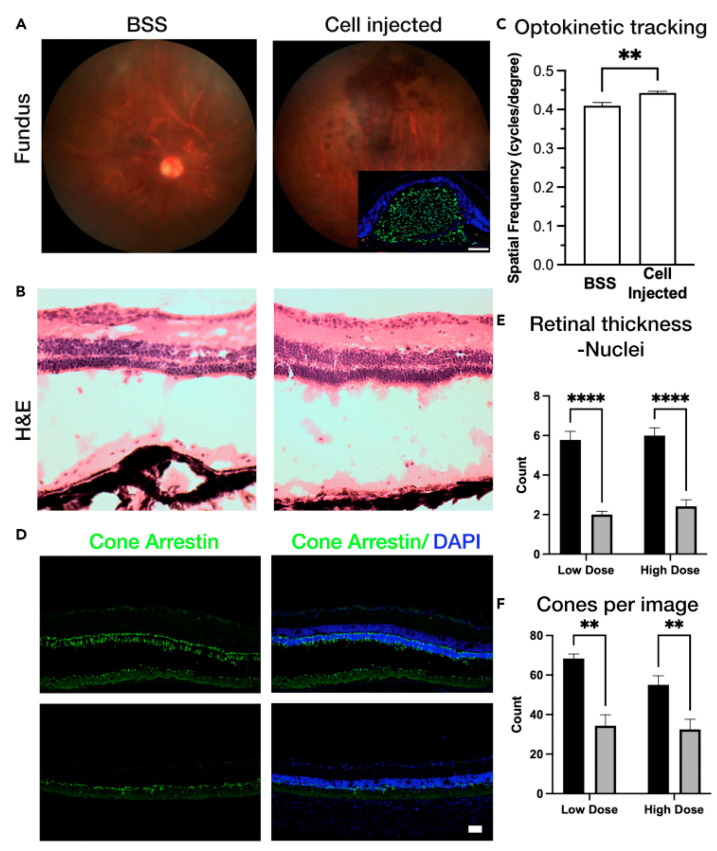
RPE cell transplantation in RCS rats. (**A**,**B**) Fundus imaging (**A**) and hematoxylin and eosin (H&E) staining (**B**) were performed on the eyes of RCS rats following subretinal injection of saline or retinal pigment epithelial (RPE) cells. The inset in (**A**) demonstrates the presence of human nuclear antigen-positive cells in the RPE-injected eye at postnatal day 90 (P90). (**C**) Assessment of visual function via optokinetic tracking revealed a significant improvement in visual acuity in RCS rats that received RPE transplantation compared to those injected with saline. (**D**) Immunohistochemical analysis of retinal sections using cone arrestin antibody was conducted to evaluate cone photoreceptor preservation following RPE transplantation. (**E**,**F**) Quantitative analysis of retinal thickness (**E**) and cone photoreceptor density (**F**) was performed in RCS rats receiving low and high doses of RPE transplantation. Measurements were taken from both the nasal and temporal regions of the retina. Scale bars represent 100 μm. Reprinted from Ref. [83].

iPSCs, classified as pluripotent stem cells, exhibit unlimited self-renewal capacity and multilineage differentiation potential [79]. iPSCs are generated through somatic cell reprogramming via ectopic expression of defined transcription factors [18]. Theoretically, their autologous origin minimizes immune rejection risks, and iPSC research has attracted increasing attention compared to ESCs in recent years. In retinal therapeutics, Akiba et al. demonstrated that human iPSCs (hiPSCs) can be differentiated into functional RPE cells with phagocytic and barrier functions [80]. D’Antonio-Chronowska et al. further developed a serum-free, small molecule-driven protocol for hiPSC-RPE differentiation, establishing a cost-effective, highly reproducible methodology enabling scalable production of RPE cells for high-throughput screening [81]. Complementing this, Truong et al. established an automated platform for hiPSC expansion and differentiation into RPE monolayers, highlighting its potential for standardized, large-scale manufacturing to address vision loss in AMD patients [82]. Figure 4 demonstrates that Surendran et al. transplanted iPSC-differentiated RPE cells into Royal College of Surgeons (RCS) rats to evaluate both the transplantation success rate and in vivo cell survival duration [83].

#### 3.1.4. MSC

MSCs, an advantageous stem cell source free from the ethical controversies associated with embryonic stem cells (ESCs), can be isolated at scale from perinatal derivatives (e.g., umbilical cord and amniotic membrane) or adult tissues (e.g., bone marrow and adipose tissue) [84]. Among MSC subtypes investigated for retinal therapeutics, bone marrow-derived MSCs (BM-MSCs) and umbilical cord-derived MSCs (UC-MSCs) are predominant. BM-MSCs, first isolated by Friedenstein et al. [85], exhibit limitations such as poor post-transplantation survival and inefficient differentiation into RPE in vivo. Huang et al. addressed these challenges by introducing ciliary neurotrophic factor (CNTF), a soluble cytokine that enhances MSC differentiation into RPE, activates photoreceptor-RPE phagocytosis, and promotes retinal repair, thereby advancing regenerative strategies [86].

**Figure 5:**
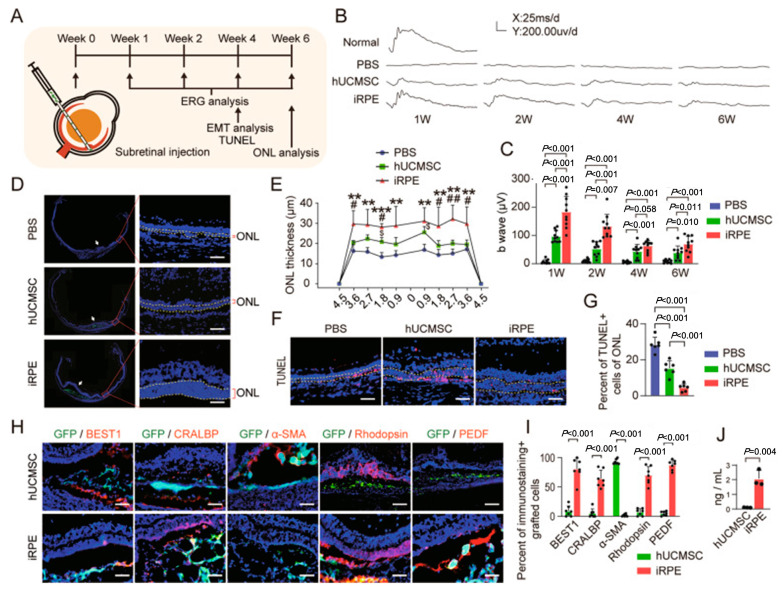
(**A**) Experimental design for the transplantation of induced retinal pigment epithelial (iRPE) cells in a sodium iodate (SI)-induced rat model of AMD. (**B**) ERG waveforms recorded at various time points post-transplantation. The calibration scale indicates 200 μV vertically and 25 ms horizontally. (**C**) Quantitative analysis of ERG b-wave amplitude (*n* = 10). Statistical significance was determined using one-way ANOVA followed by Bonferroni’s post hoc test. (**D**) Representative micrographs of retinal sections at 6 weeks post-transplantation. Injection sites are indicated by arrows, and the outer nuclear layer (ONL) is demarcated by yellow dashed lines. (**E**) Quantitative assessment of ONL thickness (μm) (*n* = 6). Statistical analysis was performed using one-way ANOVA with Bonferroni’s post hoc test. (**F**) Representative micrographs of retinal cryosections stained using the TUNEL assay to detect apoptotic cells. (**G**) Statistical analysis of the percentage of apoptotic cells within the ONL (*n* = 6). Data were analyzed using one-way ANOVA followed by Bonferroni’s post hoc test. (**H**) Immunostaining of human umbilical cord mesenchymal stem cells (hUCMSCs) and iRPE cells at 4 weeks post-transplantation in vivo. (**I**) Quantitative analysis of the percentage of immunostaining-positive grafted cells (*n* = 7). Statistical significance was determined using Student’s unpaired *t*-test. (**J**) Pigment epithelium-derived factor (PEDF) levels secreted by hUCMSCs and iRPE cells, as measured by ELISA (*n* = 4). Statistical analysis was performed using Student’s unpaired *t*-test. Scale bar = 50 μm. Data are presented as mean ± standard deviation (SD). Statistical significance is denoted as follows: ** *p* < 0.01, *** *p* < 0.001 compared to the PBS control group; ^#^ *p* < 0.05, ^##^ *p* < 0.01 compared to the hUCMSC group; ^$^ *p* < 0.05 compared to the PBS group. Adapted from Ref. [87].

UC-MSCs, in contrast, offer superior proliferative capacity, differentiation efficiency, and reduced immunogenicity compared to BM-MSCs while avoiding invasive harvesting procedures [84]. Figure 5 demonstrates that Zhu et al. established the reliable differentiation of UC-MSCs into functional RPE cells, supporting their potential as promising candidates for AMD therapy [87]. Collectively, MSCs represent a platform with substantial potential for retinal cell regeneration and AMD treatment.

#### 3.1.5. Co-Culture System for RPE

The co-culture system focuses on recreating the complex microenvironment of RPE in vivo by integrating multiple cell types (e.g., endothelial cells and photoreceptor cells), promoting the proliferation and survival of RPE, and enhancing the efficiency of the targeted induction of RPE-specific differentiation by stem cells.

Rachel et al. developed a human retinal co-culture system incorporating three cell types: human retinal microvascular endothelial cells, RPE/ARPE-19, and Müller glial cells. This tri-cellular model enhanced cellular functionality, underscored the critical role of intercellular interactions, and provided deeper mechanistic insights into retinal biology and disease pathogenesis [88]. Chang et al. explored a co-culture system utilizing human umbilical cord mesenchymal stem cells (HUCMSCs) and the human RPE-like cell line ARPE-19 to induce HUCMSC differentiation into RPE-like cells. This approach effectively promoted differentiation, with the derived cells expressing RPE-specific markers and demonstrating functional RPE characteristics, including phagocytic activity and neurotrophic factor secretion [89]. Calejo et al. developed the first co-culture model exclusively using hiPSCs as the cellular source, comprising hiPSC-derived endothelial cells (hiPSC-ECs) and hiPSC-derived retinal pigment epithelium (hiPSC-RPE). This system was cultured on opposing sides of collagen-coated porous polycaprolactone (PCL) scaffolds to establish an in vitro model mimicking the outer retinal layer. Results demonstrated the model’s utility in studying RPE-EC interactions and biomaterial-driven cellular responses. It holds potential for investigating retinal physiology/pathology and developing tissue-engineered grafts for degenerative retinal disorders [90]. These findings suggest that co-culture systems represent a promising strategy for generating RPE-like cells for AMD therapy.

### 3.2. Study of Cell-Biomaterial Scaffolds in RPE Replacement

As evidenced by prior research, cellular adhesion, proliferation, migration, and differentiation are critically governed by the physicochemical and biological properties of cell-laden biomaterial scaffolds, including mechanical stiffness, surface topography, and bioactive ligand presentation. However, subretinal delivery of these constructs necessitates invasive surgical intervention, and the therapeutic efficacy of cellular replacement remains unsubstantiated in two distinct patient cohorts: (1) individuals with early-stage AMD who decline surgery due to perceived risks of exacerbating visual morbidity and (2) patients with end-stage disease exhibiting compromised retinal architecture that precludes functional scaffold integration or host-graft crosstalk [91].

#### 3.2.1. Cell-Biomaterial Scaffold Functionality

Cell-based monotherapies face persistent challenges in achieving sufficient cell adhesion, spatiotemporal distribution, long-term survival, and functional integration at the transplantation site [92]. Consequently, researchers have shifted focus toward cell-biomaterial scaffold systems for AMD therapy, leveraging engineered scaffolds to recapitulate the retinal microenvironment and enhance therapeutic outcomes through structural, biochemical, and biomechanical support [93]. As demonstrated by Gullapalli et al. [94], injected cell suspensions exhibit suboptimal viability due to delayed scaffold adhesion (<24 h post-delivery) and susceptibility to migration, whereas biomaterial-based cell delivery systems significantly enhance survival rates through scaffold-mediated cytoprotection. This phenomenon is attributed to the scaffold’s dual role as a physicochemical niche, providing structural support for cellular anchorage, mechanotransductive signaling, and microenvironmental stabilization during integration.

#### 3.2.2. Material Properties for Cell-Biomaterial Scaffolds

Biomaterials derived from natural and synthetic polymers are currently extensively utilized as delivery scaffolds for a diverse range of retinal cells [95]. Natural polymers are extensively utilized owing to their favorable bioactivity, minimal immunogenicity, outstanding cytocompatibility, and capacity to emulate the functions of natural tissues. Nonetheless, concerns regarding the potential risk of immune rejection post-implantation, inflammatory responses, and inferior mechanical properties currently represent the primary limitations to their broader application [96]. Therefore, the physicochemical properties of these polymers are often modified by adjusting their molecular composition to meet the requirements of specific applications. To fulfill the criteria for scaffolds, the resulting cell-biomaterial scaffolds can be tailored to practical application conditions through chemical modification of natural polymers [97], blending with synthetic polymers [98], cross-linking [99], and physical modification [100]. Although synthetic polymers have excellent mechanical properties, biodegradability, and three-dimensional structures, they also have significant drawbacks, such as poor cell attachment due to the lack of bioactive sites. These limitations can be addressed through material engineering strategies, such as chemical modification [101], physical surface functionalization [102], and biomodification [103], which enhance functional performance and biocompatibility. Additionally, polymer blending (involving the combination of synthetic polymers or hybridization of natural and synthetic polymers) [104,105] and mineralization [106] provide synergistic improvements in mechanical stability and biointegration.

#### 3.2.3. Potential Solutions to the Limitations of Biomaterial Scaffolds

Biocompatibility is a critical factor in scaffold fabrication and application. By selecting appropriate materials (e.g., marine-derived collagen and alginate hydrogels) and assessing their performance through in vitro stability testing [107], in vivo experiments, and histological analyses, scaffolds can ensure efficacy and safety in tissue repair and regeneration. Biocompatible scaffolds promote tissue regeneration, reduce immune responses, and comply with ISO standards (ISO 10993-5, ISO 10993-6, and ISO 10993-11 [108,109,110]), which are essential for medical device applications [111]. These standards validate material safety for long-term implantation and subchronic toxicity.

Immune rejection remains a critical challenge for scaffold applications in tissue engineering and regenerative medicine. This issue can be mitigated by optimizing decellularization processes of biomaterials, employing immunomodulatory agents, enhancing scaffold materials and design, and implementing immune monitoring in clinical applications [112,113,114]. These strategies collectively reduce immune rejection, improve scaffold biocompatibility, and enhance functional outcomes, which are pivotal for ensuring the clinical success of tissue-engineered scaffolds [115].

Mechanical properties are crucial in scaffold production as the scaffold needs to withstand certain mechanical stresses in vivo and provide adequate mechanical support to facilitate tissue repair and regeneration. Optimizing mechanical properties can be achieved by optimizing scaffold fabrication methods, combining with other biomaterials, or undergoing chemical modifications [116,117,118]. These improvements can enhance the hardness, durability, and cell attachment ability of the scaffold. In clinical applications, the mechanical properties of the scaffold directly affect its stability and functional outcomes in vivo. Therefore, evaluating and optimizing the mechanical properties of the scaffold is key to ensuring its successful application in tissue engineering and regenerative medicine [119].

The degradation kinetics of scaffolds are a key functional indicator in tissue engineering. Excessive degradation rates may lead to premature cell migration into the surrounding matrix, compromising repair efficacy, while overly slow degradation can result in insufficient nutrient supply and physical space, ultimately hindering tissue formation [120]. Therefore, optimizing the degradation properties of scaffolds is key to ensuring their successful application in tissue engineering and regenerative medicine. This can be achieved by selecting highly degradable materials, increasing porosity, and reducing cross-linking to precisely control the degradation properties [121,122,123]. Collectively, we evaluated the comparative advantages and limitations of commonly utilized biological and synthetic polymers for fabricating cell-laden biomaterial scaffolds (Table 3) and systematically contrasted the structural characteristics of natural versus synthetic polymers with respect to biocompatibility, mechanical properties, and immunogenicity (Table 4).

## 4. Key Challenges of RPE Replacement

The primary clinical challenges associated with RPE replacement include (1) ensuring the survival of cells used for RPE transplantation [59] and assessing their long-term tissue replacement capacity after integration into the choroid [176]; (2) addressing the risk of immune rejection following cell transplantation [177]; (3) developing a safe and efficient cell-biomaterial scaffolding model based on stem cells for delivery to the retinal interstitial space [178]; (4) establishing evaluation methods to monitor RPE replacement therapy [91]; and (5) managing surgically induced damage [179].

### 4.1. Cell Graft Survival and Long-Term Replacement Capability

A critical challenge in cell replacement therapy for AMD is ensuring long-term survival and functional integration of transplanted cells. While stem cell-derived RPE grafts offer advantages such as self-renewal capacity and multipotent differentiation potential [180], achieving stable, phenotypically mature RPE populations for clinical transplantation remains problematic. Key limitations include inflammatory microenvironment-triggered apoptosis, failure of choroid-RPE complex integration, and progressive graft attrition. For instance, transplanted cells often undergo anoikis due to insufficient extracellular matrix (ECM) adhesion or immune-mediated clearance. Addressing this, Alhasani et al. demonstrated that pharmacological modulation via tauroursodeoxycholic acid (TUDCA) enhances RPE survival by attenuating oxidative stress, suppressing NLRP3 inflammasome activation, and mitigating endoplasmic reticulum stress, thereby stabilizing graft viability in preclinical models [181].

### 4.2. Host Tissue Rejection

The safety concerns that have emerged with stem cell-based therapies primarily center on cellular proliferation, the formation of ectopic tissues, and the tumorigenic potential of the injected cell mass. While these issues have not been observed in clinical studies involving autologous iPSCs, they remain a consideration for allogeneic transplants, necessitating further preclinical trials to test and enhance safety [182].

Current immunomodulatory techniques that have been developed in response to immune rejection from RPE transplantation include immunosuppressive drugs, gene editing, and encapsulation techniques. Immunosuppressive therapy is crucial for ensuring the survival and functionality of transplanted RPE cells. Drugs such as cyclosporine A, tacrolimus, and steroids can effectively suppress the activity of the immune system, reducing rejection reactions and thus enhancing the survival rate and function of the transplanted cells [183]. However, the use of these drugs needs to balance their potential side effects and requires strict monitoring and management in clinical practice. With technological advancements, some studies are exploring methods to reduce or avoid the use of immunosuppressive drugs, such as using gene-editing techniques to modify transplanted cells to improve their immune compatibility. Gene editing offers a precise approach to correct genetic mutations associated with AMD. Zhou et al. found that the decline of Klotho during aging might negatively impact retinal health, inducing age-related retinal degeneration. They used CRISPR-Cas9 gene editing technology to knock out the Klotho gene in human RPE cells, studying the role of Klotho in RPE cell function and oxidative stress. The study confirmed the protective role of Klotho in RPE cells and its impact on mitochondrial function [184]. The encapsulation technique aims to reduce the risk of immune rejection by protecting transplanted cells with a physical barrier that reduces their recognition and attack by the immune system. Encapsulation technology successfully delivers ARPE-19 cells expressing CR2-fH into mice and enables long-term secretion of CR2-fH while avoiding immune responses. This technology offers a new therapeutic strategy for AMD by systemically delivering a complement inhibitor to reduce CNV lesions [185]. However, its use in human clinical trials is still at the exploratory stage.

### 4.3. Cell Delivery

Biomaterial scaffolds, designed to support cell growth and fabricated through tissue engineering methods, represent a common approach for RPE replacement therapy. These cell-biomaterial scaffolds can address the challenges associated with traditional cell delivery methods, such as issues with cell integration, adhesion, viability, and functional polarization. However, they also face limitations due to their mechanical properties and biocompatibility. Consequently, further enhancements are necessary to apply cell-biomaterial scaffolds to RPE replacement therapies. Additionally, new tissue-engineering protocols need to be developed to improve RPE survival, adhesion, and integration and to significantly reduce the apoptosis of transplanted cells within the host tissue environment [186].

### 4.4. Evaluation of Cell Replacement

Despite successful outcomes in the follow-up of RPE transplantation after stem cell-based therapies, such as effective adherence, distribution, and long-term survival of transplanted cells at the graft site, as well as their normal functioning [92], the assessment of transplanted cell function remains crucial. Therefore, it is necessary to develop strategies capable of monitoring RPE adaptation and integration at the cellular level, accurately quantifying the amount of RPE, and evaluating its functional distribution within the retina. Moreover, reliable methods should be established to examine and characterize the improvements in AMD following RPE replacement therapy.

Optical coherence tomography (OCT) is a key imaging modality for detecting and monitoring GA due to its ability to provide detailed cross-sectional and en face images of the RPE, photoreceptor layers, and choroid. This capability is crucial for assessing disease progression and treatment efficacy in clinical trials. This is supported by the findings from the FILLY clinical trial, which demonstrated that OCT-based imaging can effectively monitor photoreceptor integrity and GA progression, highlighting its importance in evaluating therapeutic interventions [187]. Identifying biomarkers offers a precise way to assess transplantation efficacy in RPE cells. The study found that TREX, a key lncRNA, could predict and enhance transplant success, improving vision rescue in animal models [188].

### 4.5. Surgically Induced Damage

Surgery-induced injury is also a major challenge in RPE replacement therapy [179]. Conventional transplantation procedures (e.g., subretinal gap injections for RPE) can result in excessively wide incisions and a tendency for the macula (the central portion of the retina) to become fissured, leading to significant vision loss, as well as raising the risk of complications, such as immune rejection, elevated intraocular pressure, and retinal adhesion to the RPE. The risk can be reduced by preoperative injection of fibrinolytic zymogen, use of miOCT technique, customized two-hole injectors and low intraocular pressure settings, and immunosuppressive therapy [189].

The implantation of specialized devices such as biomaterial scaffolds may trigger chronic inflammatory responses or disrupt metabolic exchange between cells and the choroid [190]. Therefore, scaffolds must be nontoxic, biodegradable (to minimize foreign body residues), sufficiently thin to avoid affecting ocular focal length, mechanically robust to withstand surgical handling, and flexible to conform to retinal curvature [191,192]. However, the mechanisms and therapeutic efficacy of cell replacement remain challenging to validate, as it is unclear whether reported visual improvements arise from cell replacement, neuroprotection, or immune modulation. Thus, comprehensive long-term studies in vitro, and particularly in animal models, are essential to rigorously evaluate the biological mechanisms underlying graft-host-retina interactions [91].

### 4.6. Organoids

Retinal organoids are emerging as innovative platforms in regenerative ophthalmology, capable of recapitulating the structural and functional complexity of native retinal tissues through self-organization of pluripotent stem cells [193]. By definition, organoids possess the capacity for multi-organ lineage differentiation, mirroring the morphological architecture, tissue stratification, and physiological functionality of in vivo organs. This fidelity enables their broad utility in preclinical applications, including disease mechanism elucidation, high-throughput drug screening, personalized disease modeling, cell replacement therapy, and gene editing validation, thereby positioning them as transformative tools for advancing retinal repair strategies [194].

Building on this, the development of retinal organoids holds equally significant potential. Studies have demonstrated that both embryonic stem cells (ESCs) and induced pluripotent stem cells (iPSCs) can differentiate into three-dimensional (3D) retinal organoids [18,195]. As previously noted, RPE damage is an early occurrence in AMD [8], which is linked to the apoptosis of photoreceptors and other retinal cells [196]. The development of retinal organoids can compensate for the loss of retinal cells that results from this process. Therefore, in the future, retinal organoids are poised to become a significant avenue for the treatment of RDDs through cell transplantation. To provide a model system for the study of human photoreceptors, Cuevas et al. genetically edited NRL-deficient embryonic stem cell lines into retinal organoids [197]. Matsuyama et al. restored light responsiveness by introducing genetically engineered mouse iPSC/ESC-derived retinal patches to form synaptic connections with the host [198]. Lin et al. also explored the potential of retinal organoid transplantation to ameliorate RPE dysfunction [199]. Overall, the development of retinal organoids holds great promise. However, retinal organoids still face numerous challenges, and appropriate solutions need to be further developed to address the issues of mass production, safety, and efficacy that remain to be verified.

## 5. Conclusions

Jackson et al. identified neovascular nAMD as a principal etiology of irreversible blindness, with their STAR trial—a randomized, double-masked, sham-controlled study—demonstrating that stereotactic radiotherapy (SRT) achieved significant reduction in anti-VEGF injection frequency without compromising best-corrected visual acuity [200]. Concurrently, Liao et al. characterized GA as the advanced neurodegenerative manifestation of AMD, with their phase II trial (*n* = 246) revealing that monthly or every-other-month intravitreal pegcetacoplan administration achieved 29% reduction in lesion progression rate versus sham controls at 12-month follow-up, establishing complement pathway modulation as a viable therapeutic strategy for GA-associated visual morbidity [201]. Therefore, certain AMD subtypes constitute prevalent neurodegenerative retinal disorders and represent principal etiological factors for blindness among aging populations globally. Damage to the RPE is a key factor leading to early AMD. However, current treatments are limited in that they can only delay the progression of the disease and cannot cure permanent visual impairment. Cell-based, gene-based, and antibody-based therapies are all being trialed in the clinic, and current results suggest that these modalities have potential and need to be further explored. Recent research suggests that replacing damaged RPE is a very promising treatment, and cell-based or cell-biomaterial scaffold therapies could present a solution to this problem, but there are some major challenges associated with both of these approaches.

To date, the main challenges of cell-based or cell-biomaterial scaffold therapies include poor cell graft survival and integration, insufficient long-term replacement capacity, host rejection, difficulties in cell delivery, control of stem cell tumourigenicity, and development of systems for monitoring and evaluating cell regeneration after regeneration. Among these, cell delivery, survival, and integration issues can be addressed by developing suitable tissue-engineered cells and cell-biomaterial scaffolds and producing visual organoids. However, the tissue-engineered cell-biomaterial scaffolds also include the problems of post-implantation inflammatory response and whether the degradation time can support the survival time of RPE, while the organoids face the problems of safety, high efficiency, and mass production, and they all need to be supported by suitable protocols and complete technological methods to derive RPE cells with functionality to fully achieve the purpose of restoring visual function.

## Figures and Tables

**Table 1:** Main treatments for dry and wet AMD and their characteristics and effects.

Treatment	AMD Classification					
	Dry AMD	Characteristics and Roles	Quote	Wet AMD	Characteristics and Roles	Quote
Antibody	Lampalizumab	A humanized monoclonal antibody targeting complement factor D to inhibit alternative pathway activation in GA	[28]	Ranibizumab	Humanized monoclonal antibody fragment (anti-VEGF-A) for intravitreal injection; inhibits choroidal neovascularization in wet AMD	[29]
	Eculizumab	Humanized monoclonal anti-C5 antibody inhibiting terminal complement activation	[30]	Bevacizumab	Humanized full-length anti-VEGF monoclonal antibody used off-label intravitreally for wet AMD	[31]
	HtrA serine peptidase 1 antibody	Inhibits HtrA serine peptidase 1 activity to attenuate extracellular matrix degradation and oxidative stress in AMD	[32]	Aflibercept	VEGF receptor fusion protein; inhibits VEGF-A/B and PlGF for the treatment of wet AMD	[33]
	C3 complement inhibitor Pegcetacoplan (APL-2)	C3 complement inhibitor targeting proximal complement cascade, reducing inflammation and cellular damage in GA	[34]	Faricimab	Bispecific monoclonal antibody targeting VEGF-A and Ang-2 to treat neovascular AMD	[35]
				Brolucizumab	Humanized single-chain antibody fragment targeting VEGF-A for neovascular AMD	[36]
Gene	GT005	AAV2-based gene therapy delivering complement factor I (CFI) to suppress alternative complement pathway overactivation in geographic atrophy AMD	[37]	HMR59	AAV2 vector-based gene therapy upregulates CD59 expression on the RPE; prevents the complement cascade reaction suspected to cause macular neovascularisation	[38]
	AAVCAGsCD59	AAV-mediated gene therapy expressing soluble CD59 to inhibit complement membrane attack complex (MAC) formation in geographic atrophy AMD	[39]	ABBV-RGX-314	AAV8-based gene therapy encoding anti-VEGF Fab for neovascular AMD	[40]
	Recombinant human complement factor (GEM103)	A purified CFH replacement therapy targeting complement overactivation in geographic atrophy AMD	[41]	ADVM-022	AAV.7m8-mediated gene therapy encoding aflibercept for sustained VEGF suppression in neovascular AMD via single intravitreal injection	[42]
Cell	Human embryonic stem cell (hESC)-derived RPE	Pluripotent stem cell-derived RPE monolayers for AMD cell replacement therapy; restore retinal homeostasis via phagocytosis of photoreceptor outer segments and VEGF regulation	[43]	Coated synthetic basement membrane loaded with human ESC-derived RPE patches	Engineered Bruch’s membrane-mimetic scaffolds enhance RPE monolayer polarization and phagocytic function, improving subretinal integration in AMD models while mitigating inflammation via immunomodulatory surface coatings	[44]
	Human umbilical cord tissue-derived cells (palucorcel)	Isolated from umbilical cord tissue, exhibiting unique regenerative and immunomodulatory properties	[45]	PDMS membrane coated with laminin and liposomes loaded with dexamethasone	The PDMS membrane coated with laminin and dexamethasone-loaded liposomes integrates biomaterial engineering, extracellular matrix protein functionalization, and controlled drug delivery to achieve synergistic biological effects	[46]
	Encapsulated Cell Technology (ECT)	ECT is an advanced therapeutic platform designed for sustained intraocular drug delivery; combines cell-based bioengineering and immunoisolation strategies to enable long-term, localized secretion of therapeutic agents, addressing the limitations of conventional intravitreal injections.	[47]	Human ESC-derived RPE	Human ESC-derived RPE represents a cutting-edge regenerative approach for wet AMD, aiming to restore retinal homeostasis by replacing dysfunctional RPE cells and modulating pathological angiogenesis.	[48]
	Bone marrow-derived stem cells (BMSC)	BMSC-based therapy is a promising strategy for the treatment of dry AMD. By targeting early RPE dysfunction and blocking GA expansion, it may complement future interventions targeting photoreceptor cell regeneration, providing a comprehensive approach to this currently untreatable disease.	[49]			

**Table 2:** Main cell sources used for the treatment of AMD.

Cell Type	Cell Species	Injection Volumes/Concentrations	Injection Methods	Vantage	Challenge	Quote
RPC	Mouse	100,000 cells/µL; volume unknown	Injection into the subretinal space	Exhibits specific stem cell proliferation and differentiation properties; can differentiate into a variety of retinal cell types; lowers the risk of immune rejection and tumor development	Restricted proliferative potential; inefficient differentiation fidelity toward defined retinal neuronal lineages	[62,63,64]
ESC		150 µL of RPE cells suspension; three dose groups (1000 cells/µL, 14,000 cells/µL, 21,000 cells/µL)	Injection into the subretinal space (i.e., the anatomical interface between the atrophic photoreceptor-retinal pigment epithelium-choriocapillaris complex and the relatively preserved posterior pole retina)	Potential to differentiate into various types of cells	Ethical issues; risk of tumorigenesis; risk of immune rejection	[56,65]
iPSC	Human	2 µL of RPE cells suspension at a concentration of 2.5 × 10^4^ cells/µL in PBS	Injection into the subretinal space	Reprogrammed from adult somatic cells to avoid ethical issues; potential to differentiate into various types of cells	Tumorigenic risk; genetic or epigenetic abnormalities caused by reprogramming	[10]
MSC	Human	1 × 10^5^ cells/eye; volume unknown	Injection into the subretinal space	Derived from adult tissues, simple and widely available, immunomodulatory, low tumourigenicity	Low survival rate due to microenvironmental effects at the site of injury; further work needed to determine the best source of donors	[66,67]

**Table 3:** Biological and synthetic polymers for making cell-biomaterial scaffolds.

Polymers	Tested Species	Cell Types	Vantage	Drawbacks	Quote
Gelatine	Mouse	MSC	Biocompatibility, biodegradability, non-toxicity, plasticity, adhesion	High moisture absorption and poor mechanical properties	[124,125,126,127]
Chitosan	Mouse	RPC	Low cost, antimicrobial, low toxicity, biodegradable, biocompatible	Low solubility at physiological pH, easy interaction with other biological structures	[125,128,129,130,131,132]
Collagen	Human	RPE	Biocompatibility, biomimetic, biodegradability, haemostasis	Poor mechanical properties, poor thermal properties, enzymatic degradation	[133,134,135,136]
Alginate	Human	hESC-RPE	Easily extracted, abundantly available, biocompatible, biodegradable, nontoxic	High cost	[126,137,138,139,140]
Hyaluronic Acid	Human	RPE	Antibacterial, antioxidant, biodegradable	Readily degradable, potentially variable elements	[125,141,142,143,144,145]
Poly (lactic-co-glycolic acid) (PLGA)	Human	iPSC-RPE	Good mechanical properties, nontoxic, biodegradable, non-immunogenic, controlled drug release, biocompatible	/	[146,147,148,149,150,151]
Polycaprolactone (PCL)	Human	ARPE-19; hRPE	Biocompatible, low cost, absorbable	Insufficient mechanical strength, low number of cellular recognition sites, poor bioactivity, hydrophobicity	[134,152,153,154]
Polylactic Acid (PLA)	Human	Primary human retinal pigment epithelial cells	Biocompatibility, biodegradability, piezoelectricity	Poor mechanical properties, hydrophobicity, poor electrical conductivity	[155,156]
Parylene-C	Human	hESC-RPE; Allogeneic RPE cells	Biocompatibility, mechanical flexibility, optical transparency, low inherent stresses	Low air permeability, low mechanical strength, limited thermal budget	[157,158,159,160,161]

**Table 4:** Structured comparison of natural and synthetic polymers in terms of biocompatibility, mechanical properties, and immunogenicity.

Polymers	Biocompatible	Mechanical Properties	Immunogenicity	Quote
Gelatine	Five days post-wounding, the lightly cross-linked gelatin hydrogel significantly promotes wound healing by 60–100% and exhibits good biocompatibility.	Maximum stress in compression: 1.62–4.69 kPa; maximum stress in tension: 1.05–4.23 kPa	Mildly cross-linked gelatin hydrogel promotes cell infiltration and tissue repair without causing an immune response.	[162,163]
Chitosan	After 7 days of culture, the cell viability of hMSCs on the chitosan-containing matrix was approximately 90%, demonstrating good biocompatibility.	Elastic modulus: 2.6–12.4 kPa	1 kDa chitosan: significant anti-inflammatory effect, able to attenuate the inflammatory response by inducing Tregs; thus, its molecular weight has an important influence on its immunomodulatory properties.	[164,165]
Collagen	The number of L929 cells increased by a factor of 3.5–4 within 7 days, demonstrating good biocompatibility.	Elastic modulus: 1–20 kPa	Although collagen is biocompatible, xenogeneic collagen has potential for immune reactions.	[166]
Alginate	The viability of ARPE-19 cells was significantly increased (*p* < 0.05), demonstrating good biocompatibility.	Elevated oxidant ratio and reduced viscosity	Downregulated inflammation and upregulated expression of anti-inflammatory cytokines	[137,167]
Hyaluronic Acid	The expression of ARPE-19-specific proteins and genes was significantly increased (*p* < 0.05), demonstrating good biocompatibility.	Inherent mechanical properties are not strong but can be enhanced by chemical modification and cross-linking.	Does not cause an immune response	[145,168]
Poly (lactic-co-glycolic acid) (PLGA)	Frequently utilized as drug delivery vehicles, it exhibits biocompatibility.	Elastic modulus: 15 ± 3–150 ± 38 MPa	FDA- and EMA-approved drug delivery system for parenteral administration that does not cause an immune response	[169]
Polycaprolactone (PCL)	The viability of porcine islet cells was significantly increased (93.8% ± 2.7%, *p* < 0.05), demonstrating good biocompatibility.	Elastic modulus: 195–531 MPa	PCL scaffolds elicited a weaker immune response in animal models, as evidenced by less immune cell infiltration and a lower inflammatory response.	[170,171]
Polylactic Acid (PLA)	The increased number of natural killer cells on the PLA scaffold indicates good biocompatibility.	Elastic modulus: 1.91 ± 0.09 GPa	After PLA implantation, there is a mild inflammatory response that gradually decreases as the PLA degrades.	[172,173,174]
Parylene-C	L929 cells were co-cultured with eluates extracted from parylene-C-coated chips for 24 h, showing a cytotoxicity grade of 0.	Reduced tensile strength (not specified)	Bioinsulation assessment and hypersensitivity assessment showed that Parylene-C has good biocompatibility and insulating properties, with an overall favorable immune response.	[158,175]

## Data Availability

Data sharing is not applicable to this article as no datasets were generated or analyzed during the current study.
